# From the ECM to the Cytoskeleton and Back: How Integrins Orchestrate T Cell Action

**DOI:** 10.1155/2000/86281

**Published:** 2000

**Authors:** Jennifer A. Epler, Rugao Liu, Yoji Shimizu

**Affiliations:** 1 Department of Laboratory Medicine and Pathology Center for Immunology Cancer Center University of Minnesota Medical Minneapolis MN 55455 USA; 2 Department of Laboratory Medicine and Pathology University of Minnesota Medical School Box 334 Mayo/312 Church St. SE Minneapolis MN 55455 USA

## Abstract

T lymphocytes constitute a highly dynamic tissue type. During the course of their lives, they travel through a variety of physiological environments and experience a multitude of interactions with extracellular matrix components and other cells. In order to do this, they must receive many environmental cues, and translate these signals into the appropriate biological actions. Particularly dramatic are the cytoskeletal shape changes a T cell must undergo during the processes of leaving the bloodstream, migrating through tissues, and encountering antigen. In this review, we highlight the role of integrins in providing a link between the extracellular environment and cytoskeletal regulation and how these receptors help to orchestrate T cell migration and antigen recognition.


*Developmental Immunology*, 2000, Vol. 7(2-4), pp. 155-170
Reprints available directly from the publisher
Photocopying permitted by license only

© 2000 OPA (Overseas Publishers Association)
N.V. Published by license under the
Harwood Academic Publishers imprint,
part of the Gordon and Breach Publishing Group.
Printed in Malaysia

### From the ECM to the Cytoskeleton and Back: How Integrins Orchestrate T Cell Action

JENNIFER A. EPLER, RUGAO LIU and YOJI SHIMIZU*

*Department of Laboratory Medicine and Pathology, Center for Immunology, Cancer Center, University of Minnesota Medical School, Minneapolis, MN 55455*

T lymphocytes constitute a highly dynamic tissue type. During the course of their lives, they travel through a variety of physiological environments and experience a multitude of interactions with extracellular matrix components and other cells. In order to do this, they must receive many environmental cues, and translate these signals into the appropriate biological actions. Particularly dramatic are the cytoskeletal shape changes a T cell must undergo during the processes of leaving the bloodstream, migrating through tissues, and encountering antigen. In this review, we highlight the role of integrins in providing a link between the extracellular environment and cytoskeletal regulation and how these receptors help to orchestrate T cell migration and antigen recognition.

Integrin cell surface receptors are heterodimeric α/β pairs that have extracellular matrix (ECM) components as well as other cell surface proteins in their ligand repertoire. An array of structurally distinct α and β subunits combine to form over 20 distinct integrin receptors. Integrin subunits are characterized by large extracellular domains and comparatively short cytoplasmic tails, with the notable exception of β4, which has a large cytoplasmic domain. The β2 integrin subfamily, which includes the LFA-1 (αLβ2), Mac-1 (αMβ2) and p150,95 (αXβ2) integrins, the β1 integrin subfamily, which includes the α4β1 and α5β1 integrins, and the α4β7 integrin play particularly notable roles in T cell function. The relevance of integrins to a variety of biological processes is illustrated by their ubiquitous expression on all nucleated cells and the dramatic effects of genetic ablation of most integrin α and β subunits in mice (Clark and Brugge, 1995; Schwartz et al., 1995; Shimizu et al., 1999; Hynes and Bader, 1997; Hynes, 1996).

Several aspects of integrin structure and function lead to their prominent role in regulating the ability of a T cell to interact with and respond to the extracellular environment. First, the short cytoplasmic tails associate with cytoskeletal proteins, such as talin, α-actinin and paxillin (Clark and Brugge, 1995). In this way, integrins act as a cell surface bridge that links the structure of the extracellular matrix environment around a cell with its own cytoskeletal scaffold. In adherent cells, this linkage with the cytoskeleton results in the formation of a structure known as a focal adhesion at the point of contact of a cell with the underlying ECM (Schwartz et al., 1995; Guan, 1997). In addition to providing cell anchorage, focal adhesions are now known to be centers of signaling activity. Kinases, such as src kinase and focal adhesion

* Address correspondence to: Yoji Shimizu, Ph.D., Department of Laboratory Medicine and Pathology, University of Minnesota Medical School, Box 334 Mayo/312 Church St. SE, Minneapolis, MN 55455. TEL: 612–626–6849; FAX: 612–625–2199; E-mail: shimi002@tc.umn. edu kinase (FAK), as well as adapter proteins, localize in focal adhesions (Guan, 1997). Although lymphocytes do not form classical focal adhesions (Serrador et al., 1999), integrin linkage to the cytoskeleton plays an equally critical role in regulating T cell function.

A second aspect of integrin function that is critical to orchestration of T cell action is the ability of T cells to dynamically regulate the functional activity of integrins in response to environmental cues (Diamond and Springer, 1994; Shimizu and Hunt, III, 1996). Thus, integrins can cycle between different states of activity that consequently alter T cell adhesiveness to the ECM, and to cells expressing integrin counter-receptors. These changes may involve alterations in the conformation of integrin extracellular domains that result in increased ligand binding affinity, as well as cytoskeleton-dependent changes in the localization of integrins on the cell surface that result in increased avidity (Diamond and Springer, 1994; Bazzoni and Hemler, 1998). The dynamic nature of these changes in integrin activity allows for the precise regulation of T cell interactions with the ECM and with other cells. These responses are necessary for appropriate migration and responses to antigen.

A final integrin function that is critical to T cell action is the ability of integrins to transduce intracellular signals upon ligand engagement (Clark and Brugge, 1995; Schwartz et al., 1995). In adherent cells, integrin signaling plays a central role in regulating integrin-dependent cell migration (Guan, 1997; Schlaepfer et al., 1999), as well as providing signals that insure cell survival upon anchorage to the ECM (Clark and Brugge, 1995; Giancotti, 1997). Although T cells do not exhibit a similar requirement for ECM attachment in order to survive, integrin signaling does promote T cell proliferation (Shimizu et al., 1990a; Udagawa et al., 1996; Geginat et al., 1999). In addition, the highly migratory lifestyle of a T cell suggests a central role for integrin signaling in regulating T cell movement.

The initiation of an antigen-specific T cell response requires that T cells move out of the bloodstream into secondary lymphoid tissues or sites of inflammation, migrate through these tissues, and interact with antigen-presenting cells (APCs). T cells face a formidable task in achieving the morphological changes necessary for this characteristic travel. In this review, we highlight recent insights into the role of integrins and the ECM in each of these stages of T cell action (Figure 1).

### LEAVING THE BLOODSTREAM

Integrins figure prominently in the ability of T cells to traffic to different sites around the body (Butcher et al., 1999). Different integrins help to determine different routes, and promote different stages of travel. The route covered by circulating naïve T lymphocytes is limited and relatively uncomplicated, covering the blood stream and secondary lymphoid tissue, such as lymph nodes. Memory/effector T cells cover a much more diverse area as they carry out surveillance functions. They may enter non-lymphoid tissues, such as the skin, and can also travel the same routes covered by naïve cells.

The specificity in the routes of migration of T cells is orchestrated in large part by the interplay of adhesion receptors, endothelial cell substrata and chemokines. This interplay determines the ability of an individual T cell to extravasate at a specific site. Lymphocyte extravasation involves three successive steps: (1) primary adhesion of lymphocytes to the endothelium, which is manifested as rolling or tumbling under shear flow conditions; (2) lymphocyte activation, which results in integrin-dependent stable arrest on endothelium; and (3) transmigration of lymphocytes out of the blood stream to lymphoid tissues or inflammation sites (diapedesis) (Butcher et al., 1999; Springer, 1995; Butcher, 1991). The interaction between integrins and other adhesion receptors with their ECM or cell surface ligands plays an essential role in each step of the extravasation phase.

### Primary Adhesion

Although selectin-mediated adhesion to carbohydrate based ligands plays a prominent role in lymphocyte tethering and rolling on the venular endothelium

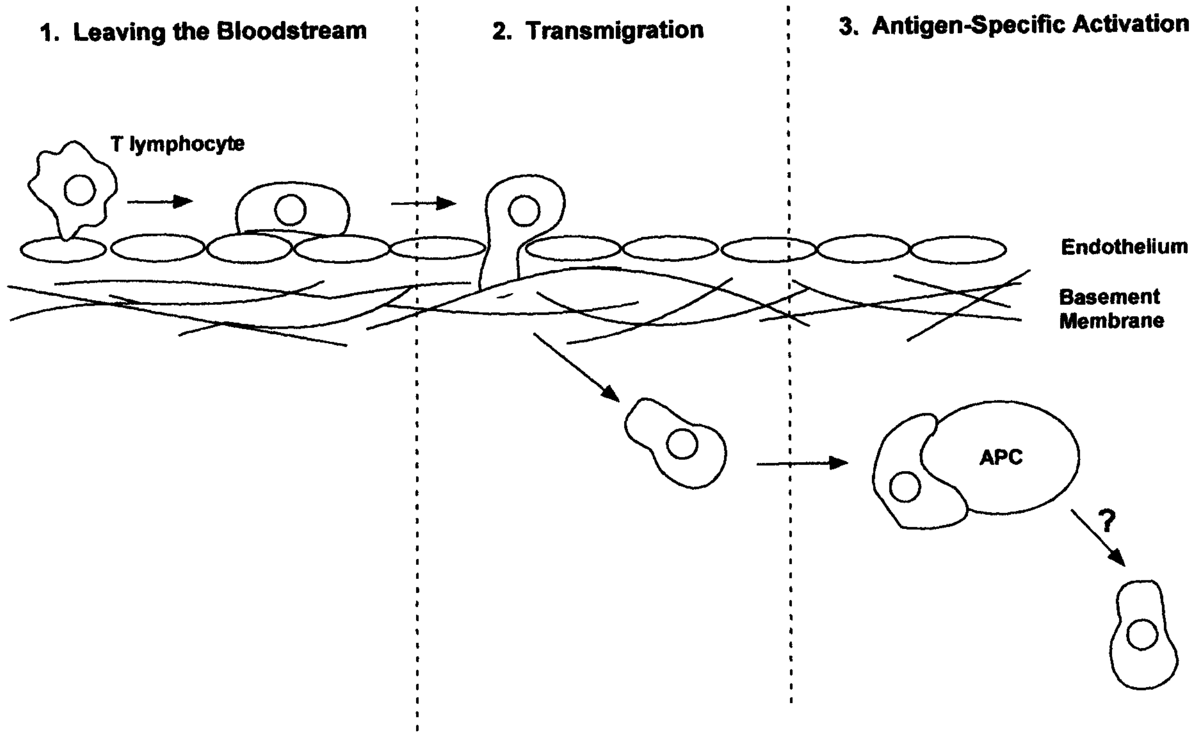

FIGURE 1 Integrins and T cell action. A T cell must invoke many shape changes in order to the leave the bloodstream (1), migrate through tissue (2), and respond to antigen (3). Integrins figure prominently in the ability of the T cell cytoskeletal architecture to evoke these changes

(Butcher et al., 1999; Lawrence et al., 1995; Alon et al., 1994), the α4β1 and α4β7 integrins can also mediate this initial step in leukocyte extravasation (Berlin et al., 1995; Berlin et al., 1993; Sriramarao et al., 1994; Alon et al., 1995). Like L-selectin, α4β7 is highly concentrated on the microvilli of lymphocytes (Berlin et al., 1995). This places α4β7 in a region of the cell surface that is critical in initiating lymphocyte contact with endothelium under shear flow conditions. This tethering interaction allows sufficient time for a circulating lymphocyte to retrieve information from the endothelial surface, most notably the presence (or absence) of a signal capable of activating integrins and initiating stable, shear-resistant attachment.

### Activating Integrins: The Role of Chemokines

Chemokines (chemotactic cytokines) are a large group of low molecular weight secretory or membrane bound proteins that provide directional cues for lymphocyte migration (Ward et al., 1998; Baggiolini, 1998; Kim and Broxmeyer, 1999). Several lines of evidence strongly suggest that one function of chemokines in lymphocyte extravasation is to provide an activating signal to integrins, resulting in shear-resistant attachment to endothelium. First, the ability of pertussis toxin to block lymphocyte extravasation (Bargatze and Butcher, 1993; Bargatze et al., 1995) is consistent with the interaction of chemokines with pertussis toxin-sensitive G protein-coupled receptors. Second, numerous chemokines can rapidly increase integrin-mediated adhesion of lymphocytes. In vitro, RANTES, MCP-1 and MCP-β all induce β1 integrin-dependent T lymphocyte adhesion to fibronectin (Carr et al., 1996). SDF-1α, SDF-1β, MIP-3β and 6-C-kine all trigger rapid and transient adhesion of human lymphocytes to ICAM-1 via β2 integrins (Campbell et al., 1998). Third, chemokines can be detected on endothelial surfaces, which is where they must reside in order to activate rolling lymphocytes. For example, 6-C-kine, which is a potent chemoattractant for naïve T cells, is detectable on high endothelial venules found in peripheral lymph nodes (Gunn et al., 1998; Willimann et al., 1998). This is consistent with a proposed role for 6-C-kine in triggering integrin activation during naïve T cell interactions with peripheral lymph node HEV. Fourth, loss of chemokine expression in mice can disrupt lymphocyte trafficking. Notably, naïve T cells do not migrate to peripheral lymph nodes in mice lacking 6-C-kine (Gunn et al., 1999), which is consistent with a critical role for 6-C-kine in mediating T cell trafficking into peripheral lymph nodes. Thus, the spectrum of chemokines produced in a local endothelial area, coupled with the spectrum of chemokine receptors expressed by any given T cell, likely determines the efficiency of integrin activation during interactions with endothelium.

The mechanism by which chemokines are "presented" to rolling lymphocytes is critical, since chemokines must achieve a local threshold concentration in order to activate integrins expressed on T cells. This would be difficult to accomplish with chemokines in solution, since they would be rapidly diluted and swept away once secreted into the blood vessel. It is now clear that chemokines can overcome this problem by binding to cell surfaces (Tanaka et al., 1993b). In vitro studies have shown that integrin-dependent adhesion of T cells can be triggered by chemokines that are immobilized via their heparin-binding properties to proteoglycans and glycosaminoglycans (Tanaka et al., 1993a; Gilat et al., 1996; Gilat et al., 1994). In addition, studies with IL-8 have suggested that chemokines may accumulate at membrane protrusions on endothelial cells, increasing the their local concentration (Rot et al., 1996; Middleton et al., 1997). The chemokine fractalkine represents a novel member of the chemokine family that is expressed on cell surfaces by a direct transmembrane linkage. This allows for presentation on the cell surface by a stalk-like extracellular domain (Bazan et al., 1997). Thus, chemokines are likely to be specifically retained on the endothelial cell substrata, resulting in local availability of chemokines to rolling lymphocytes at concentrations sufficiently high enough to result in integrin activation (Witt and Lander, 1994). Specific anatomic "conduits" have also been proposed to serve as a mechanism by which to direct chemokines to HEVs in lymph nodes (Gretz et al., 1996; Ebnet et al., 1996). Furthermore, binding of chemokines to ECM components is likely to play a role in the development of chemokine gradients that are essential for directed lymphocyte migration (Gilat et al., 1996; Lider et al., 1995).

### *Chemokine signaling and integrin activation*

The biological effects of chemokines are mediated by their interaction with serpentine G-protein-coupled receptors (Ward et al., 1998). Despite the well-appreciated role of chemokines in regulating integrin-dependent adhesion and migration, little is still known regarding the biochemical events that mediate chemokine-induced integrin activation. Although chemokine-induced triggering of calcium mobilization is well established (Ward et al., 1998), its role in regulating integrin function is undefined. Chemokines also trigger tyrosine phosphorylation events (Ward et al., 1998), but again, the relationship of these biochemical events to integrin activation has not been established. The small GTP-binding protein, Rho, has been implicated in integrin activation by chemokines, based on the ability of C3 transferase exoenzyme to block chemokine-induced activation of $\alpha 4\beta 1$ (Laudanna et al., 1996). These findings suggest that Rho participates in a signal cascade from the chemokine receptor to trigger integrin-mediated lymphocyte adhesion. Although the pathways linking chemokine receptors to Rho activation are not fully elucidated for lymphocytes, PKC$\zeta$ seems to be involved in integrin activation induced by fMLP in human leukocytes (Laudanna et al., 1998). Although a role for the lipid kinase phosphoinositide 3-OH kinase (PI 3-K) in the regulation of integrin function by immunoglobulin superfamily members has been established (Shimizu and Hunt, III, 1996), PI 3-K inhibitors do not block integrin activation by fMLP in human neutrophils (Jones et al., 1998).

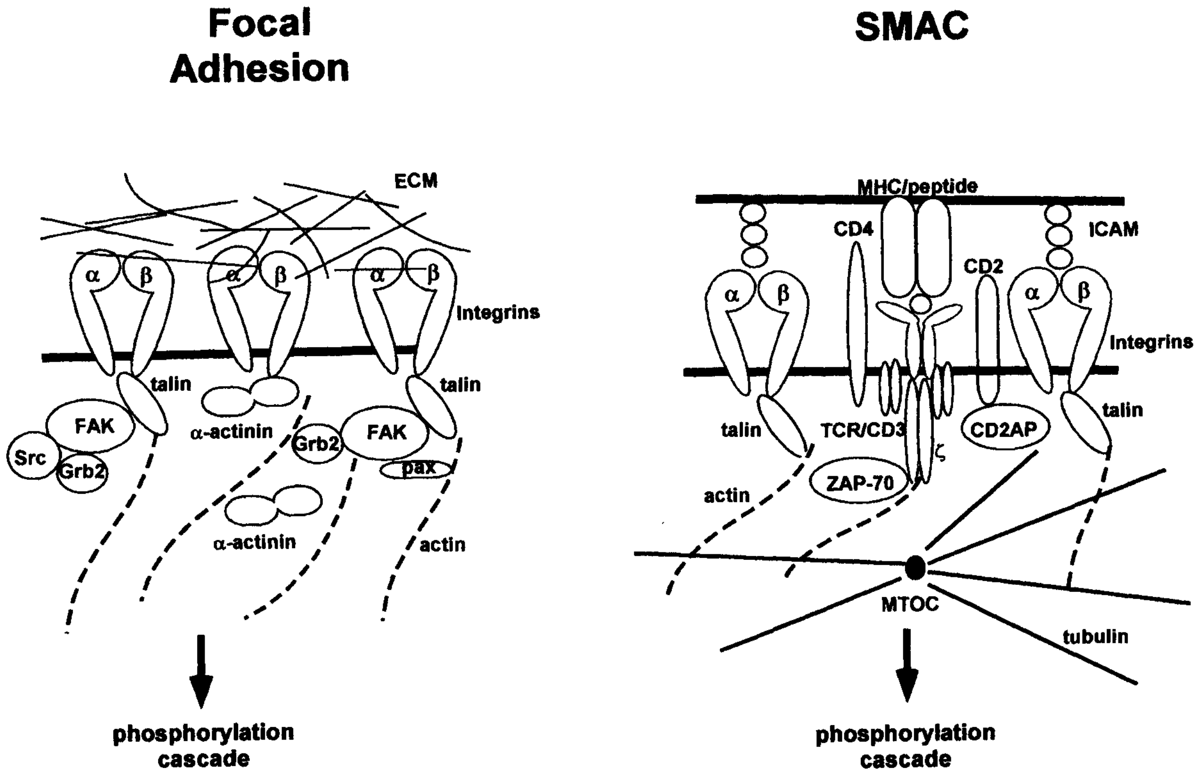

FIGURE 2 Focal adhesions and SMACs represent areas of coordinated signal transduction. The focal adhesions studied extensively in fibroblasts bear a striking resemblance to the SMACs formed in T cells. Both occupy a significant area of cell membrane and include integrin receptor clustering. Talin and polymerized actin are associated with integrin β cytoplasmic tails in each. Focal adhesions and SMACs represent origins of extensive biochemical signaling cascades that impact cell growth and differentiation

### Are There Other Mechanisms by which Integrins are Activated During Extravasation?

In addition to chemokines, other receptors may play a role in initiating intracellular signals that activate integrins during interactions with endothelium. Ligation of L-selectin results in increased integrin-mediated adhesion (Hwang et al., 1996; Giblin et al., 1997; Steeber et al., 1997), suggesting that selectin-mediated rolling leads to signals resulting in shear-resistant attachment mediated by integrins. In vitro studies have also suggested a role for CD31 in integrin activation, as well as transendothelial migration (Tanaka et al., 1992; Schimmenti et al., 1992; Bogen et al., 1992). However, T lymphocyte homing is normal in CD31-deficient mice (Duncan et al., 1999). Integrins themselves may also play a role in regulating the activity of other integrins, a regulatory phenomenon referred to as integrin "cross-talk" (Blystone et al., 1994; Porter and Hogg, 1998; Porter and Hogg, 1997). Studies with T cells have shown that interaction of LFA-1 with ICAM-1 inhibits α4β1 integrin-mediated adhesion but enhances T cell migration on fibronectin. Since chemokines can also differentially modulate the activity of β1 and β2 integrins on T cells (Carr et al., 1996), the temporal regulation of distinct integrin types may be critical to successful lymphocyte extravasation. This model also predicts that the spectrum of integrin ligands expressed on a given endothelial surface is likely to play a critical role in regulating lymphocyte extravasation. For example, VCAM-1 has been proposed to be expressed primarily on inflamed endothelium, although recent studies have detected a low level of basal expression of VCAM-1 on lymph node HEV, as well as a role for α4β1 and α4β7 in T cell interactions with lymph node HEV (Berlin-Rufenach et al., 1999). Surface-associated fibronectin expressed on endothelial surfaces has also been proposed to play a role in lymphocyte interactions with endothelium (Szekanecz et al., 1992; Ager, 1997).

### LYMPHOCYTE TRANSMIGRATION

In vitro studies have implicated both α4β1 and LFA-1 in transendothelial migration (Oppenheimer-Marks et al., 1991; Butcher et al., 1999; Oppenheimer-Marks et al., 1990; Brezinschek et al., 1995). In addition, a role for αvβ3 in monocyte transmigration has been proposed (Weerasinghe et al., 1998). The differential and sequential activation of integrin receptors determines the program of temporal and spatial coordination of cell adhesion, spreading and migration in lymphocytes.

The major morphological changes that occur during lymphocyte transmigration include cell spreading in response to activation signals, which results in shear-resistant attachment, and the induction of cell motility. This results in movement of lymphocytes through the endothelial monolayer and into the underlying basement membrane. Cytoskeletal reorganization provides a driving force for cell spreading and migration. The small GTP binding proteins of the Rho family are key regulators of cellular morphology (Hall, 1998; Reif and Cantrell, 1998). In fibroblasts, different members of the Rho family have distinct effects on cell morphology. While Rho acts as a molecular switch to regulate the assembly of focal adhesion complexes and contractile actin-myosin filaments, Rac activation leads to the assembly of meshworks of actin filaments at the periphery to produce lamellipodia and membrane ruffles. Cdc42 activity results in the formation of filopodia, actin-rich cell surface protrusions. In T lymphocytes, expression of an active form of Rac increases α4β1- and α5β1 integrin-mediated cell adhesion and spreading (D'Souza-Schorey et al., 1998), suggesting a possible role for Rac in morphological changes that are required for lymphocyte transmigration. The role of Rho in chemokine signaling in leukocytes (Laudanna et al., 1996) is also consistent with a function for these GTP-binding proteins in lymphocyte transmigration.

Initiation of cell locomotion requires a membrane protrusion at the leading edge (Serrador et al., 1999). Actin and actin binding proteins such as paxillin and vinculin, along with integrin receptors and kinases are concentrated at the leading edge. In addition, chemokine receptors accumulate at the front portion of migratory cells (Serrador et al., 1999). After the formation and stabilization of the leading edge, cells use myosin-based proteins to generate contractile action and force for cell movement (Serrador et al., 1998). A distinct structure known as a uropod forms at the trailing end of migrating lymphocytes and contains actin binding proteins as well as the cell surface receptors ICAM-1, –2 and –3, CD43 and CD44 (Serrador et al., 1998). In addition to the adhesive force provided by integrins during cell motility, signaling initiated by integrin engagement by ligand regulates cell migration. Studies of adherent cells have implicated FAK in regulating integrin-dependent cell migration, since over-expression of FAK enhances cell migration (Cary et al., 1996) and FAK-deficient fibroblasts show reduced migration when compared to wild-type fibroblasts (Ilic et al., 1995). The effects of FAK on cell migration may be mediated by downstream tyrosine phosphorylation of the adapter protein $p130^{Cas}$ (Cary et al., 1998; Klemke et al., 1998) as well as the Crk adapter protein (Klemke et al., 1998). Although β1 integrin-mediated tyrosine phosphorylation of FAK in adherent cells has been clearly established, there are conflicting reports on the ability of β1 integrins on T cells to initiate tyrosine phosphorylation of FAK (Nojima et al., 1995; Maguire et al., 1995; Hunter and Shimizu, 1997). Thus, the role of FAK in regulating T cell migration remains an area for further exploration. However, both β1 and β2 integrins expressed on lymphocytes induce tyrosine phosphorylation of $p130^{Cas}$ and a structurally related protein, HEF1 or Cas-L (Petruzzelli et al., 1996;

Hunter and Shimizu, 1997; Minegishi et al., 1996). PI 3-K has been implicated in regulating integrin-dependent motility of tumor cells in response to growth factor stimulation (Shaw et al., 1997; Adelsman et al., 1999; Adam et al., 1998). In addition, activation of mitogen-activated protein kinase (MAPK) has been suggested to play a role in regulating cell migration via phosphorylation of myosin light chain kinase (Klemke et al., 1997). Enhancement of COS cell migration is also observed following over-expression of ICAP-1 (Zhang and Hemler, 1999), an intracellular protein that associates with the β1 integrin cytoplasmic domain (Chang et al., 1997). It is currently unclear whether these additional pathways of regulating cell migration are also operative in T cells during the transmigration process.

Mechanisms by which integrin receptor activity is inhibited must also be invoked, given findings that active integrin receptors are localized at the leading edge and inactive ones are found at the trailing portion of migrating cells (Serrador et al., 1999). In addition to integrin cross talk mechanisms that allow some integrins to inhibit others (Porter and Hogg, 1997; Blystone et al., 1999), a number of intracellular molecules can negatively regulate integrin-mediated cell adhesion. Overexpression of integrin-linked kinase (ILK), a molecule that was initially identified based on its association with integrin β subunit cytoplasmic domains, decreases β1 integrin mediated adhesion of human epithelial cells (Hannigan et al., 1996). A novel expression genetic strategy has also demonstrated a role for active H-ras in suppressing integrin function (Hughes et al., 1997). More recently, expression of PTEN, a tumor suppressor with lipid and protein phosphatase activity, was shown to inhibit cell spreading, focal adhesion and migration (Tamura et al., 1998). The role of these molecules in regulating T cell motility has not been extensively investigated. In hematopoietic cells, tyrosine phosphatases play a central role in negatively regulating integrin-mediated adhesion. T cell lines deficient in expression of the CD45 tyrosine phosphatase show enhanced β1 integrin-dependent adhesion to fibronectin (Shenoi et al., 1999), and studies with macrophages deficient in expression of the SHP-1 phosphatase show that SHP-1 is required for detachment from adhesion mediated by the αMβ2 integrin (Roach et al., 1998).

### Regulation of ECM-degrading Proteases by Integrins

Not only must T cells squeeze through the endothelial monolayer, they must also have mechanisms for breaking through the underlying basement membrane. This complex of various ECM proteins, such as laminin and type IV collagen, constitutes a rigid barrier. It has been shown in a variety of cell types that integrins have the additional function of inducing the expression of specialized proteases on the surface of migrating cells (Romanic and Madri, 1994; Huhtala et al., 1995; Brooks et al., 1996). These proteases are of the matrix metalloproteinase (MMP) family, which are specifically designed for ECM protein degradation (Huhtala et al., 1995). Interaction of T cells with VCAM-1 via α4β1 results in surface expression of the MMP family member, 72 kD gelatinase (Romanic and Madri, 1994). In addition, the 72 kD gelatinase inhibitor, TIMP-2, blocks in vitro T cell transmigration, suggesting a critical role for α4β1 integrin-mediated induction of expression of this protease in T cell migration.

Interestingly, there is evidence in fibroblast studies of a role for integrin cross-talk in MMP induction. Engagement of α5β1 results in increased MMP expression, while α4β1 integrin stimulation produces only low basal expression. Simultaneous engagement of α5β1 and α4β1 also results in low expression of MMPs (Huhtala et al., 1995). Whether these specific integrin roles, or the general cross talk phenomenon, applies to surface MMP induction in T cells remains to be seen.

The activity of membrane-bound proteases must be subject to strict regional control. The active protease must sense the leading edge of the cell, and be present only in this area, and in a matrix protein-specific manner. In addition to regulating protease expression, integrins also participate in regional control of protease expression on the cell surface. Studies with CS-1 melanoma cells have revealed the colocalization of αVβ3 integrin and MMP-2 on the cell surface that is mediated by direct binding of MMP-2 to αVβ3 itself (Brooks et al., 1996). This association implies a distinct and directed method by which tumor cells invade specific tissues. The intriguing possibility of similar MMP-integrin juxtapositions in T cells is suggested.

### INTEGRINS AND ANTIGEN-SPECIFIC T CELL ACTIVATION

Although T lymphocytes exhibit high rates of migration (Serrador et al., 1999), antigen recognition by T lymphocytes in lymphoid tissue requires that antigen-specific T cells be detained long enough within the tissue site for activation and differentiation to occur. Integrins also participate in this complex process of antigen-specific T cell activation, and many of the same regulatory events that govern cell migration also govern the changes that occur in T cells as a result of encounter with antigen.

### Stop Signals

The conversion from a migratory to a more stationary phenotype in T cells is mediated by engagement of the antigen-specific T cell receptor (TCR) with peptide antigen presented by a self-MHC protein on a tissue-resident APC. In vitro studies have shown that TCR stimulation results in two distinct changes in integrin-dependent function. One is a rapid, but transient increase in β1- and β2 integrin-mediated adhesion of T cells to ECM proteins such as fibronectin and laminin, as well as cell surface counter-receptors such as ICAM-1 and VCAM-1 (Dustin and Springer, 1989; van Kooyk et al., 1989; Shimizu et al., 1990b). This effect of TCR stimulation is similar in some respects to the effects of chemokines on integrin function under conditions of shear flow. TCR-induced increases in β 1 integrin-mediated adhesion of T cells to ECM proteins may be particularly critical in providing a mechanism by which to retain antigen-reactive T cells at the site of antigen encounter. A second effect of TCR stimulation is to block T cell migration on purified ICAM-1 (Dustin et al., 1997). Treatment of T cells with antibodies that induce the high affinity conformation of LFA-1 can also induce this "stop signal", suggesting that TCR stimulation induces this block in migration by inducing an increase in LFA-1 affinity (Dustin et al., 1997). However, an ability of TCR stimulation to induce changes in LFA-1 affinity has not been uniformly observed (Stewart et al., 1996). Nevertheless, initial TCR engagement leads to changes in integrin function that result in dramatic effects on T cell adhesion and migration.

### Morphological and Cytoskeletal Changes Upon APC Engagement

As the TCR recognizes its peptide antigen in the clutches of the appropriate MHC receptor on the APC, it turns its full attention to the site of recognition. The accessory proteins CD4/8 cluster about the engaged TCR, stabilizing the interaction. CD4 and CD8 recognize conserved sites on the MHC protein (class II or I) and recruit critical cytoplasmic signaling proteins (eg., $p56^{lck}$) into the vicinity of the TCR. Co-receptors that provide amplifying signals to TCR activation, such as CD28, also likely are recruited to the site of contact between the TCR and APC. Early studies with blocking antibodies against LFA-1 and CD2 demonstrated a critical role for these adhesion molecules in mediating conjugate formation between T cells and target cells (Shaw et al., 1986). During this process of APC engagement, signals provided by the TCR and co-receptors, such as CD2 and CD28, serve to stabilize the T cell-APC interaction by enhancing the functional activity of β2, as well as β1, integrins. Stimulation of CD2, CD28 or CD7 can enhance integrin-mediated adhesion, even in the absence of simultaneous engagement of the TCR (van Kooyk et al., 1989; Shimizu et al., 1990b; Shimizu et al., 1992). This suggests that a critical function of co-receptor signaling during T cell activation is to enhance integrin-mediated adhesive forces that are necessary for effective stimulation of T cells (Zell et al., 1998a). Receptors that activate integrin-mediated adhesion also activate PI 3-K, and studies with pharmacological and genetic inhibitors of PI 3-K show a clear role for PI 3-K in the activation of integrin function by the TCR, CD2, CD7, and CD28 (Nagel et al., 1998; Chan et al., 1997; Shimizu et al., 1995; Zell et al., 1998b; Kivens et al., 1998; Zell et al., 1996). For LFA-1, TCR-induced increases in LFA-1-mediated adhesion to ICAM-1 may involve an intracellular protein, cytohesin-1, that associates with the β2 integrin cytoplasmic domain and is a downstream target of PI 3-K (Nagel et al., 1998; Kolanus et al., 1996).

Recent studies of APC engagement have vividly demonstrated the formation of a specialized structure in the T cell at the point of contact with the APC termed a "supramolecular activation cluster" (SMAC) (Monks et al., 1998) or "immunological synapse" (Shaw and Dustin, 1997). This bull's eye-shaped structure consists of an inner circle, or central SMAC (cSMAC), that contains a tight cluster of specific and co-operative signaling molecules, such as CD4 and the TCR on the surface, and $p56^{lck}$, $p59^{fyn}$ and PKC θ in the proximal cytoplasm (Monks et al., 1998). Complementary studies utilizing an alternative approach with purified adhesion molecules suggest that CD2 is also found in the cSMAC (Dustin et al., 1998). The outer ring, the peripheral SMAC (pSMAC), is defined by the presence of LFA-1 and the integrin-cytoskeletal linker protein, talin (Monks et al., 1998). Based on the extracellular "heights" of these proteins (shorter-in-the-middle, longer-on-the-edge) a concave 3D structure (Shaw and Dustin, 1997) is formed. Thus, LFA-1, which provides much of the adhesive force between T cells and APCs during this process, forms a ring of adhesion around smaller receptors that mediate lower affinity interactions.

Complementing the SMAC arrangement on the surface is a strikingly similar cytoskeletal rearrangement. Upon TCR engagement, the microtubule organizing center (MTOC) moves from the vicinity of the uropod to the point directly below the TCR (Serrador et al., 1999; Sedwick et al., 1999). Simultaneously, the actin cytoskeleton rearranges to form an asymmetric cap structure centered around the MTOC (Sedwick et al., 1999; Holsinger et al., 1998; Serrador et al., 1999). Interestingly, the translocation of actin and the microtubule structures appears to be independent of each other, and dependent upon signals from different cell surface receptors. Despite evidence for actin cap formation by T cells placed on anti-CD3 coated plates (Holsinger et al., 1998), more recent data suggests that MTOC relocation is the distinct result of TCR engagement, while actin capping (as measured by localization of the actin binding protein talin) is the result of LFA-1 binding. This independence was demonstrated in a novel experiment in which T cells were exposed to antigen-free, ICAM-expressing APC and anti-CD3 coated beads from opposite poles (Sedwick et al., 1999). Talin and actin polarized at the site of LFA-1/ICAM binding at the APC, whereas the MTOC translocated to the site of bead-cell contact.

In many respects, SMACs are strikingly similar to focal adhesions created by integrins in adherent cell types (Guan, 1997) (Figure 2). Both SMACs and focal adhesions are marked by extensive receptor clustering, which leads to the initiation of diverse intracellular signaling events. In addition, the cytoskeleton plays a central role in the formation of both structures. In the case of SMACs the interaction of talin with the β2 cytoplasmic domain may be particularly important, since mutations in the β2 cytoplasmic domain have dramatic effects on LFA-1 function (Hibbs et al., 1991). However, it has not yet been demonstrated that LFA-1 interactions with talin are required for SMAC formation. Studies of SMAC formation with T cells lacking LFA-1 may be particularly informative regarding the precise role of integrins in the formation and maintenance of SMACs during T cell activation.

### Lipid Rafts and SMACs?

Lipid rafts is a term used to define regions of the T cell membrane that consist of detergent-resistant zones enriched in cholesterol and sphingolipids, as well as a variety of key signaling proteins (Xavier et al., 1998; Moran and Miceli, 1998). Intact lipid rafts are required for efficient T cell signal transduction (Moran and Miceli, 1998; Xavier et al., 1998; Stulnig et al., 1999) and T cell stimulation with beads containing anti-CD3 and anti-CD28 mAbs results in polarization of lipid rafts to the point of contact between the T cell and the bead (Viola et al., 1999).

Src family tyrosine kinases, certain PI 3-K isoforms, and adapter proteins such as LAT (*l*inker for *a*ctivation of *T* cells) are enriched in the lipid rafts (Zhang et al., 1998; Xavier et al., 1998; Harder and Simons, 1999), consistent with a role for these structures in T cell activation. Recently, it has been suggested that aggregation of lipid rafts at the T cell-APC zone of contact is associated with actin cytoskeleton reorganization, as disruption of the rafts inhibits the association of signaling proteins, such as TCR ζ, with the cytoskeleton. However, the mechanism of promotion remains unknown (Moran and Miceli, 1998). Because lipid rafts also localize to the T cell-APC contact zone and have a provocative connection to the cytoskeleton, they have a striking resemblance both to SMACs and focal adhesions. However, the relationship between these biochemically defined raft regions of the T cell plasma membrane and the microscopically defined SMACs remains unclear. In particular, the localization of integrins to lipid rafts remains an unexplored area.

### TCR Signaling and Cytoskeletal Reorganization

Signals transduced by the TCR have now been linked to intracellular events that lead to reorganization of the cytoskeleton. Tyrosine phosphorylation of the ζ tail by $p56^{lck}$ induces the association of TCR ζ with the actin cytoskeleton (Rozdzial et al., 1995; Rozdzial et al., 1998). In addition, the immunoreceptor tyrosine-based activation motifs (ITAMs) in the TCR ζ cytoplasmic domain are important in TCR-driven reorientation of the microtubule organizing center and polymerization of the actin cytoskeleton (Lowin-Kropf et al., 1998; Rozdzial et al., 1998). Since tyrosine phosphorylation of ITAMs results in the association and activation of the ZAP-70 tyrosine kinase, a role for ZAP-70 and its downstream substrates in regulating the cytoskeleton upon TCR stimulation would be predicted. Recent studies have confirmed this hypothesis. Overexpression of a dominant negative form of ZAP-70 in T cells prevents MTOC reorganization (Lowin-Kropf et al., 1998). In addition, ZAP-70-mediated tyrosine phosphorylation of the adapter protein SLP-76 is critical to the formation of a trimolecular complex consisting of tyrosine phosphorylated SLP-76, Vav and Nck (Wardenburg et al., 1998). This complex results in the recruitment via Nck of p21-activated protein kinase 1 (PAK1), a kinase that has been implicated in actin polymerization and that is activated by GTP-bound Rac and Cdc42 (Sells et al., 1997; Adam et al., 1998). Since Vav functions as a GDP-GTP exchanger for Rho family proteins, including Cdc42 and Rac, PAK is activated in this complex due to its proximity to GTP-bound Rac and Cdc42. The ability of dominant-negative forms of SLP-76, Vav and Nck to inhibit TCR-induced polymerization of the actin cytoskeleton (Wardenburg et al., 1998)is consistent with a role for this trimolecular complex in regulating TCR-driven cytoskeletal rearrangement, possibly via PAK1 or another protein that interacts with Nck, such as Wiskott-Aldrich syndrome protein (Ramesh et al., 1999; Bi and Zigmond, 1999). Independent studies with Vav-deficient T cells have also demonstrated a role for Vav in the induction of actin caps following stimulation with immobilized anti-CD3 mAbs (Holsinger et al., 1998; Fischer et al., 1998; Cantrell, 1998). However, the precise relationship of these biochemical events to the SMAC formation or polarization of lipid rafts remains to be determined.

### Co-receptor Signaling and the T Cell Cytoskeleton

The role of integrins in regulating the cytoskeleton during the process of antigen-specific T cell activation remains poorly characterized. However, both β1 and β2 integrins can enhance TCR-driven T cell proliferation (Shimizu et al., 1990a; van Seventer et al., 1990; Udagawa et al., 1996; Abraham et al., 1999), suggesting that integrin signaling might contribute to the cytoskeletal rearrangements that are required for T cell activation. Recent studies demonstrating a role for LFA-1-dependent cell spreading in facilitating TCR-driven activation of MAPK is consistent with this notion (Geginat et al., 1999). However, the spatial segregation in SMACs of LFA-1 from other receptors that participate in T cell activation, such as the TCR itself, CD2 and CD28, suggests that there may be unique features of integrin signaling as it relates to its downstream effects on T cells. Other co-receptors that promote T cell proliferation clearly can initiate signals that result in cytoskeletal rearrangements. CD28 stimulation leads to activation of PAK1 that can be enhanced by simultaneous engagement of the TCR (Kaga et al., 1998a), and CD28 signaling also leads to an increase in F-actin content in T cells (Kaga et al., 1998b). Engagement of CD2 leads to reorientation of the MTOC that is regulated by a novel intracellular protein, CD2AP, that associates with the CD2 cytoplasmic domain (Dustin et al., 1998).

### Localization of β1 Integrins During T Cell Activation

Although LFA-1 has been localized to pSMACs, the redistribution of β1 and β7 integrins during antigen-specific T cell activation remains unknown. This is a critical issue for several reasons. First, VCAM-1 has been detected on certain antigen-presenting cells, including follicular dendritic cells (Ogata et al., 1996; Gao et al., 1997). Thus, the potential exists for α4β1, and possibly α4β7, to mediate adhesion between antigen-specific T cells and certain APCs. Second, signals provided by the ECM via β1 integrins can enhance TCR-induced T cell proliferation and induction of gene expression (Shimizu et al., 1990a; Udagawa et al., 1996). The localization of β1 integrins in either SMACs or lipid rafts is likely to provide important insights into the biochemical basis and functional outcomes of ECM-mediated signals that impinge on antigen-specific T cell activation.

### TERMINATING THE T CELL-APC CONTACT

The mechanistic basis for the termination of the interaction between an antigen-specific T cell and an antigen-laden APC remains unclear, although this process of termination is clearly critical to the dissemination of effector T cells through the body following antigen challenge. Biochemical mechanisms that downregulate integrin function are likely to play a critical role in this termination event. Processes that downregulate integrins during cell motility might also participate in downregulating integrin function during T cell activation. Recent findings that CD45 and other tyrosine phosphatases may have a negative regulatory effect on integrins are particularly intriguing (Shenoi et al., 1999; Roach et al., 1998), given the vital role that CD45 plays in T cell activation in general.

### IL-2 and T Cell Migration

Although the role of IL-2 in promoting T cell proliferation is well appreciated, this cytokine may also play an important role in regulating T cell motility during antigen challenge. IL-2 stimulation leads to the transcription of a number of cytoskeletal proteins, including β-catenin, β-actin and α-tubulin. These transcriptional events may be related to the increase in T cell size that occurs during T cell activation (Herblot et al., 1999). IL-2 also enhances T cell adhesion to fibronectin, laminin and type-IV collagen, as well as fibronectin-dependent migration in vitro (Ariel et al., 1998). Interestingly, naturally occurring IL-2 fragments produced by the neutrophil enzyme, elastase, can alter these pro-adhesion and pro-migration abilities (Ariel et al., 1998). However, the prevalence of these IL-2 fragments in vivo is still uncertain. IL-2 also induces actin polymerization and membrane ruffling in human and mouse T cells (Arrieumerlou et al., 1998). Many intracellular signaling events that regulate integrin function, such as activation of PI 3-K and phosphorylation of the phosphatase SHP-2, occur upon engagement of the IL-2 receptor (Arrieumerlou et al., 1998; Brennan et al., 1997; González-Garcia et al., 1997). How these IL-2 receptor-mediated signals integrate with signals provided by the TCR, integrins and other co-receptors to regulate T cell adhesion and motility remain important areas of future investigation.

### CONCLUSION

Each step of a T cell's journey through lymphoid and remote tissues depends on integrin activity. Not only does this activity exert concerted homing effects, it is also a critical component of the cytoskeletal regulation physically necessary for motion through diverse environments. Although much of our knowledge of how integrins and the cytoskeleton influence cell motility results from studies in non-lymphoid cells, it is becoming clear that lymphocytes utilize many of these same regulatory mechanisms. In addition, it is apparent that integrins participate in the development of novel specialized structures, such as SMACs, that are critical for antigen-specific T cell activation. Our understanding of T lymphocyte action will continue to be enriched by further examination of the dynamic, integrin-mediated actions immediately preceding and distantly following antigen recognition.

### *Acknowledgements*

Supported by NIH grants AI31126 and AI38474, and by Department of Defense grant DAMD17–97–1–7228. R.L. is supported by a postdoctoral trainee award provided by NIH CA09138. Y.S. is the Harry Kay Chair of Cancer Research at the University of Minnesota.

### *References*

Abraham, C., Griffith, J., and Miller, J. (1999). The dependence for leukocyte function-associated antigen-1/ICAM-1 interactions in T cell activation cannot be overcome by expression of high density TCR ligand. J. Immunol. **162**:4399–4405.

Adam, L., Vadlamudi, R., Kondapaka, S.B., Chernoff, J., Mendelsohn, J., and Kumar, R. (1998). Heregulin regulates cytoskeletal reorganization and cell migration through the p21-activated kinase-1 via phosphatidylinositol-3 kinase. J. Biol. Chem. **273**:28238–28246.

Adelsman, M.A., McCarthy, J.B., and Shimizu, Y. (1999). Stimulation of β1 integrin function by epidermal growth factor and heregulin-β has distinct requirements for erbB2 but a similar dependence on PI 3-kinase. Mol. Biol. Cell **10**:2861-2878.

Ager, A. (1997). Regulation of lymphocyte migration into lymph nodes by high endothelial venules. Biochem. Soc. Trans. **25**:421–428.

Alon, R., Kassner, P.D., Carr, M.W., Finger, E.B., Hemler, M.E., and Springer, T.A. (1995). The integrin VLA-4 supports tethering and rolling in flow on VCAM-1. J. Cell Biol. **128**:1243–1254.

Alon, R., Rossiter, H., Wang, X., Springer, T.A., and Kupper, T.S. (1994). Distinct cell surface ligands mediate T lymphocyte attachment and rolling on P and E selectin under physiological flow. J. Cell Biol. **127**:1485–1495.

Ariel, A., Yavin, E.J., Hershkoviz, R., Avron, A., Franitza, S., Hardan, I., Cahalon, L., Fridkin, M., and Lider, O. (1998). IL-2 induces T cell adherence to extracellular matrix: inhibition of adherence and migration by IL-2 peptides generated by leukocyte elastase. J. Immunol. **161**:2465–2472.

Arrieumerlou, C., Donnadieu, E., Brennan, P., Keryer, G., Bismuth, G., Cantrell, D., and Trautmann, A. (1998). Involvement of phosphoinositide 3-kinase and Rac in membrane ruffling induced by IL-2 in T cells. Eur. J. Immunol. **28**:1877–1885.

Baggiolini, M. (1998). Chemokines and leukocyte traffic. Nature **392**:565–568.

Bargatze, R.F. and Butcher, E.C. (1993). Rapid G protein-regulated activation event involved in lymphocyte binding to high endothelial venules. J. Exp. Med. **178**:367–372.

Bargatze, R.F., Jutila, M.A., and Butcher, E.C. (1995). Distinct roles of L-selectin and integrin α4β7 in lymphocyte homing to Peyer's patch-HEV in situ: the multistep model confirmed and refined. Immunity **3**:99–108.

Bazan, J.F., Bacon, K.B., Hardiman, G., Wang, W., Soo, K., Rossi, D., Greaves, D.R., Zlotnik, A., and Schall, T.J. (1997). A new class of membrane-bound chemokine with a $CX_3C$ motif. Nature **385**:640–644.

Bazzoni, G. and Hemler, M.E. (1998). Are changes in integrin affinity and conformation overemphasized. Trends Biochem. Sci. **23**:30–34.

Berlin-Rufenach, C., Otto, F., Mathies, M., Westermann, J., Owen, M.J., Hamann, A., and Hogg, N. (1999). Lymphocyte migration in lymphocyte function-associated antigen (LFA)-1-deficient mice. J. Exp. Med. **189**:1467–1478.

Berlin, C., Bargatze, R.F., Campbell, J.J., Von Andrian, U.H., Szabo, M.C., Hasslen, S.R., Nelson, R.D., Berg, E.L., Erlandsen, S.L., and Butcher, E.C. (1995). α4 integrins mediate lymphocyte attachment and rolling under physiologic flow. Cell **80**:413–422.

Berlin, C., Berg, E.L., Briskin, M.J., Andrew, D.P., Kilshaw, P.J., Holzmann, B., Weissman, I.L., Hamann, A., and Butcher, E.C. (1993). α4β7 integrin mediates lymphocyte binding to the mucosal vascular addressin MAdCAM-1. Cell **74**:185–195.

Bi, E.F. and Zigmond, S.H. (1999). Actin polymerization: where the WASP stings. Curr. Biol. **9**:R160-R163.

Blystone, S.D., Graham, I.L., Lindberg, F.P., and Brown, E.J. (1994). Integrin $\alpha_v\beta_3$ differentially regulates adhesive and phagocytic functions of the fibronectin receptor $\alpha_5\beta_1$. J. Cell Biol. **127**:1129–1137.

Blystone, S.D., Slater, S.E., Williams, M.P., Crow, M.T., and Brown, E.J. (1999). A molecular mechanism of integrin cross-talk: $\alpha_v\beta_3$ suppression of calcium/calmodulin-dependent protein kinase II regulates $\alpha_5\beta_1$function. J. Cell Biol. **145**:889–897.

Bogen, S.A., Baldwin, H.S., Watkins, S.C., Albelda, S.M., and Abbas, A.K. (1992). Association of murine CD31 with transmigrating lymphocytes following antigenic stimulation. Am. J. Pathol. **141**:843–854.

Brennan, P., Babbage, J.W., Burgering, B.M.T., Groner, B., Reif, K., and Cantrell, D.A. (1997). Phosphatidylinositol 3-kinase couples the interleukin-2 receptor to the cell cycle regulator E2F. Immunity **7**:679–689.

Brezinschek, R.I., Lipsky, P.E., Galea, P., Vita, R., and Oppenheimer-Marks, N. (1995). Phenotypic characterization of $CD4^+$ T cells that exhibit a transendothelial migratory capacity. J. Immunol. **154**:3062–3077.

Brooks, P.C., Strömblad, S., Sanders, L.C., Von Schalscha, T.L., Aimes, R.T., Stetler-Stevenson, W.G., Quigley, J.P., and Cheresh, D.A. (1996). Localization of matrix metalloproteinase MMP-2 to the surface of invasive cells by interaction with integrin αvβ3. Cell **85**:683–693.

Butcher, E.C. (1991). Leukocyte-endothelial cell recognition: three (or more) steps to specificity and diversity. Cell **67**:1033–1036.

Butcher, E.C., Williams, M., Youngman, K., Rott, L., and Briskin, M. (1999). Lymphocyte trafficking and regional immunity. Adv. Immunol. **72**:209–253.

Campbell, J.J., Hedrick, J., Zlotnik, A., Siani, M.A., Thompson, D.A., and Butcher, E.C. (1998). Chemokines and the arrest of lymphocytes rolling under flow conditions. Science **279**:381–384.

Cantrell, D. (1998). Lymphocyte signalling: a coordinating role for Vav? Curr. Biol. **8**:R535-R538.

Carr, M.W., Alon, R., and Springer, T.A. (1996). The C-C chemokine MCP-1 differentially modulates the avidity of β 1 and β2 integrins on T lymphocytes. Immunity **4**:179–187.

Cary, L.A., Chang, J.F., and Guan, J.L. (1996). Stimulation of cell migration by overexpression of focal adhesion kinase and its association with Src and Fyn. J. Cell Sci. **109**:1787–1794.

Cary, L.A., Han, D.C., Polte, T.R., Hanks, S.K., and Guan, J.L. (1998). Identification of $p130^{Cas}$as a mediator of focal adhesion kinase-promoted cell migration. J. Cell Biol. **140**:211–221.

Chan, A.S.H., Mobley, J.L., Fields, G.B., and Shimizu, Y. (1997). CD7-mediated regulation of integrin adhesiveness on human T cells involves tyrosine phosphorylation-dependent activation of phosphatidylinositol 3-kinase. J. Immunol. **159**:934–942.

Chang, D.D., Wong, C., Smith, H., and Liu, J. (1997). ICAP-1, a novel $\beta_1$ integrin cytoplasmic domain-associated protein, binds to a conserved and functionally important NPXY sequence motif of $\beta_1$ integrin. J. Cell Biol. **138**:1149–1157.

Clark, E.A. and Brugge, J.S. (1995). Integrins and signal transduction pathways: the road taken. Science **268**:233–239.

D'Souza-Schorey, C., Boettner, B., and Van Aelst, L. (1998). Rac regulates integrin-mediated spreading and increased adhesion of T lymphocytes. Mol. Cell. Biol. **18**:3936–3946.

Diamond, M.S. and Springer, T.A. (1994). The dynamic regulation of integrin adhesiveness. Curr. Biol. **4**:506–517.

Duncan, G.S., Andrew, D.P., Takimoto, H., Kaufman, S.A., Yoshida, H., Spellberg, J., De la Pompa, J.L., Elia, A., Wakeham, A., Karan-Tamir, B., Muller, W.A., Senaldi, G., Zukowski, M.M., and Mak, T.W. (1999). Genetic evidence for functional redundancy of platelet/endothelial cell adhesion molecule-1 (PECAM-1): CD31-deficient mice reveal PECAM-1-dependent and PECAM-1 -independent functions. J. Immunol. **162**:3022–3030.

Dustin, M.L., Bromley, S.K., Kan, Z.Y., Peterson, D.A., and Unanue, E.R. (1997). Antigen receptor engagement delivers a stop signal to migrating T lymphocytes. Proc. Natl. Acad. Sci. USA **94**:3909–3913.

Dustin, M.L., Olszowy, M.W., Holdorf, A.D., Li, J., Bromley, S., Desai, N., Widder, P., Rosenberger, F., Van der Merwe, P.A., Allen, P.M., and Shaw, A.S. (1998). A novel adaptor protein orchestrates receptor patterning and cytoskeletal polarity in T-cell contacts. Cell **94**:667–677.

Dustin, M.L. and Springer, T.A. (1989). T-cell receptor cross-linking transiently stimulates adhesiveness through LFA-1. Nature **341**:619–624.

Ebnet, K., Kaldjian, E.P., Anderson, A.O., and Shaw, S. (1996). Orchestrated information transfer underlying leukocyte endothelial interactions. Annu. Rev. Immunol. **14**:155–177.

Fischer, K.D., Kong, Y.Y., Nishina, H., Tedford, K., Marengère, L.E., Kozieradzki, I., Sasaki, T., Starr, M., Chan, G., Gardener, S., Nghiem, M.P., Bouchard, D., Barbacid, M., Bernstein, A., and Penninger, J.M. (1998). Vav is a regulator of cytoskeletal reorganization mediated by the T-cell receptor. Curr. Biol. **8**:554–562.

Gao, J.X., Madrenas, J., Zeng, W., Zhong, R., and Grant, D. (1997). Generation of dendritic cell-like antigen-presenting cells in long-term mixed leucocyte culture: phenotypic and functional studies. Immunology **91**:135–144.

Geginat, J., Bossi, G., Bender, J.R., and Pardi, R. (1999). Anchorage dependence of mitogen-induced $G_1$to S transition in primary T lymphocytes. J. Immunol. **162**:5085–5093.

Giancotti, F.G. (1997). Integrin signaling: specificity and control of cell survival and cell cycle progression. Curr. Opin. Cell Biol. **9**:691–700.

Giblin, P.A., Hwang, S.T., Katsumoto, T.R., and Rosen, S.D. (1997). Ligation of L-selectin on T lymphocytes activates $\beta_1$ integrins and promotes adhesion to fibronectin. J. Immunol. **159**:3498–3507.

Gilat, D., Cahalon, L., Hershkoviz, R., and Lider, O. (1996). Interplay of T cells and cytokines in the context of enzymatically modified extracellular matrix. Immunol. Today **17**;16–20.

Gilat, D., Hershkoviz, R., Mekori, Y.A., Vlodavsky, I., and Lider, O. (1994). Regulation of adhesion of $CD4^+$ T lymphocytes to intact or heparinase-treated subendothelial extracellular matrix by diffusible or anchored RANTES and MIP-1β. J. Immunol. **153**:4899–4906.

González-García, A., Mérida, I., Martinez, C., and Carrera, A.C. (1997). Intermediate affinity interleukin-2 receptor mediates survival via a phosphatidylinositol 3-kinase-dependent pathway. J. Biol. Chem. **272**:10220–10226.

Gretz, J.E., Kaldjian, E.P., Anderson, A.O., and Shaw, S. (1996). Sophisticated strategies for information encounter in the lymph node – the reticular network as a conduit of soluble information and a highway for cell traffic. J. Immunol. **157**:495–499.

Guan, J.L. (1997). Focal adhesion kinase in integrin signaling. Matrix Biol. **16**:195–200.

Gunn, M.D., Kyuwa, S., Tam, C., Kakiuchi, T., Matsuzawa, A., Williams, L.T., and Nakano, H. (1999). Mice lacking expression of secondary lymphoid organ chemokine have defects in lymphocyte homing and dendritic cell localization. J. Exp. Med. **189**:451–460.

Gunn, M.D., Tangemann, K., Tam, C., Cyster, J.G., Rosen, S.D., and Williams, L.T. (1998). A chemokine expressed in lymphoid high endothelial venules promotes the adhesion and chemotaxis of naive T lymphocytes. Proc. Natl. Acad. Sci. USA **95**:258–263.

Hall, A. (1998). Rho GTPases and the actin cytoskeleton. Science **279**:509–514.

Hannigan, G.E., Leung-Hagesteijn, C., Fitz-Gibbon, L., Coppolino, M.G., Radeva, G., Filmus, J., Bell, J.C., and Dedhar, S. (1996). Regulation of cell adhesion and anchorage-dependent growth by a new $\beta_1$-integrin-linked protein kinase. Nature **379**:91–96.

Harder, T. and Simons, K. (1999). Cluster of glycolipid and glycosylphosphatidylinositol-anchored proteins in lymphoid cells: accumulation of actin regulated by local tyrosine phosphorylation. Eur. J. Immunol. **29**:556–562.

Herblot, S., Chastagner, P., Samady, L., Moreau, J.-L., Demaison, C., Froussard, P., Liu, X., Bonnet, J., and Thèze, J. (1999). IL-2-dependent expression of genes involved in cytoskeleton organization, oncogene regulation, and transcriptional control. J. Immunol. **162**:3280–3288.

Hibbs, M.L., Xu, H., Stacker, S.A., and Springer, T.A. (1991). Regulation of adhesion to ICAM-1 by the cytoplasmic domain of LFA-1 integrin β subunit. Science **251**:1611–1613.

Holsinger, L.J., Graef, I.A., Swat, W., Chi, T., Bautista, D.M., Davidson, L., Lewis, R.S., Alt, F.W., and Crabtree, G.R. (1998). Defects in actin-cap formation in Vav-deficient mice implicate an actin requirement for lymphocyte signal transduction. Curr. Biol. **8**:563–572.

Hughes, P.E., Renshaw, M.W., Pfaff, M., Forsyth, J., Keivens, V.M., Schwartz, M.A., and Ginsberg, M.H. (1997). Suppression of integrin activation: a novel function of a Ras/Raf-initiated MAP kinase pathway. Cell **88**:521–530.

Huhtala, P., Humphries, M.J., McCarthy, J.B., Tremble, P.M., Werb, Z., and Damsky, C.H. (1995). Cooperative signaling by α5β and α4β1 integrins regulates metalloproteinase gene expression in fibroblasts adhering to fibronectin. J. Cell Biol. **129**:867–879.

Hunter, A.J. and Shimizu, Y. (1997). α4β1-mediated tyrosine phosphorylation in human T cells: characterization of Crk- and Fyn-associated substrates (pp105, pp115, and human enhancer of filamentation-1) and integrin-dependent activation of $p59^{fyn}$. J. Immunol. **159**:4806–4814.

Hwang, S.T., Singer, M.S., Giblin, P.A., Yednock, T.A., Bacon, K.B., Simon, S.I., and Rosen, S.D. (1996). GlyCAM-1, a physiologic ligand for L-selectin, activates β2 integrins on naive peripheral lymphocytes. J. Exp. Med. **184**:1343–1348.

Hynes, R.O. (1996). Targeted mutations in cell adhesion genes: what have we learned from them. Dev. Biol. **180**:402–412.

Hynes, R.O. and Bader, B.L. (1997). Targeted mutations in integrins and their ligands: their implications for vascular biology. Thromb. Haemost. **78**:83–87.

Ilic, D., Furuta, Y., Kanazawa, S., Takeda, N., Sobue, K., Nakatsuji, N., Nomura, S., Fujimoto, J., Okada, M., Yamamoto, T., and Aizawa, S. (1995). Reduced motility and enhanced focal adhesion contact formation in cells from FAK-deficient mice. Nature **377**:539–544.

Jones, S.L., Knaus, U.G., Bokoch, G.M., and Brown, E.J. (1998). Two signaling mechanisms for activation of αMβ2 avidity in polymorphonuclear neutrophils. J. Biol. Chem. **273**:10556–10566.

Kaga, S., Ragg, S., Rogers, K.A., and Ochi, A. (1998a). Activation of p21-CDC42/Rac-activated kinases by CD28 signaling: p21-activated kinase (PAK) and MEK kinase 1 (MEKK1) may mediate the interplay between CD3 and CD28 signals. J. Immunol. **160**:4182–4189.

Kaga, S., Ragg, S., Rogers, K.A., and Ochi, A. (1998b). Cutting edge: Stimulation of CD28 with B7-2 promotes focal adhesion-like cell contacts where Rho family small G proteins accumulate in T cells. J. Immunol. **160**:24–27.

Kim, C.H. and Broxmeyer, H.E. (1999). Chemokines: signal lamps for trafficking of T and B cells for development and effector function. J. Leukocyte Biol. **65**:6–15.

Kivens, W.J., Hunt, S.W., III, Mobley, J.L., Zell, T., Dell, C.L., Bierer, B.E., and Shimizu, Y. (1998). Identification of a proline-rich sequence in the CD2 cytoplasmic domain critical for regulation of integrin-mediated adhesion and activation of phosphoinositide 3-kinase. Mol. Cell. Biol. **18**:5291–5307.

Klemke, R.L., Cai, S., Giannini, A.L., Gallagher, P.J., de Lanerolle, P., and Cheresh, D.A. (1997). Regulation of cell motility by mitogen-activated protein kinase. J. Cell Biol. **137**:481–492.

Klemke, R.L., Leng, J., Molander, R., Brooks, P.C., Vuori, K., and Cheresh, D.A. (1998). CAS/Crk coupling serves as a "molecular switch" for induction of cell migration. J. Cell Biol. **140**:961–972.

Kolanus, W., Nagel, W., Schiller, B., Zeitlmann, L., Godar, S., Stockinger, H., and Seed, B. (1996). αLβ2 integrin/LFA-1 binding to ICAM-1 induced by cytohesin- 1, a cytoplasmic regulatory molecule. Cell **86**:233–242.

Laudanna, C., Campbell, J.J., and Butcher, E.C. (1996). Role of Rho in chemoattractant-activated leukocyte adhesion through integrins. Science **271**:981–983.

Laudanna, C., Mochly-Rosen, D., Liron, T., Constantin, G., and Butcher, E.C. (1998). Evidence of ζ protein kinase C involvement in polymorphonuclear neutrophil integrin-dependent adhesion and chemotaxis. J. Biol. Chem. **273**:30306–30315.

Lawrence, M.B., Berg, E.L., Butcher, E.C., and Springer, T.A. (1995). Rolling of lymphocytes and neutrophils on peripheral node addressin and subsequent arrest on ICAM-1 in shear flow. Eur. J. Immunol. **25**:1025–1031.

Lider, O., Hershkoviz, R., and Kachalsky, S.G. (1995). Interactions of migrating T lymphocytes, inflammatory mediators, and the extracellular matrix. Crit. Rev. Immunol. **15**:271–283.

Lowin-Kropf, B., Shapiro, V.S., and Weiss, A. (1998). Cytoskeletal polarization of T cells is regulated by an immunoreceptor tyrosine-based activation motif-dependent mechanism. J. Cell Biol. **140**:861–871.

Maguire, J.E., Danahey, K.M., Burkly, L.C., and van Seventer, G.A. (1995). T cell receptor- and β1 integrin-mediated signals synergize to induce tyrosine phosphorylation of focal adhesion kinase ($pp125^{FAK}$) in human T cells. J. Exp. Med. **182**:2079–2090.

Middleton, J., Neil, S., Wintle, J., Clark-Lewis, I., Moore, H., Lam, C., Auer, M., Hub, E., and Rot, A. (1997). Transcytosis and surface presentation of IL-8 by venular endothelial cells. Cell **91**:385–395.

Minegishi, M., Tachibana, K., Sato, T., Iwata, S., Nojima, Y., and Morimoto, C. (1996). Structure and function of Cas-L, a 105-kD Crk-associated substrate-related protein that is involved in β1 integrin- mediated signaling in lymphocytes. J. Exp. Med. **184**:1365–1375.

Monks, C.R.F., Freiberg, B.A., Kupfer, H., Sciaky, N., and Kupfer, A. (1998). Three-dimensional segregation of supramolecular activation clusters in T cells. Nature **395**:82–86.

Moran, M. and Miceli, M.C. (1998). Engagement of GPI-linked CD48 contributes to TCR signals and cytoskeletal reorganization: a role for lipid rafts in T cell activation. Immunity **9**:787–796.

Nagel, W., Zeitlmann, L., Schilcher, P., Geiger, C., Kolanus, J., and Kolanus, W. (1998). Phosphoinositide 3-OH kinase activates the $\beta_2$ integrin adhesion pathway and induces membrane recruitment of cytohesin-1. J. Biol. Chem. **273**:14853–14861.

Nojima, Y., Tachibana, K., Sato, T., Schlossman, S.F., and Morimoto, C. (1995). Focal adhesion kinase ($pp125^{FAK}$) is tyrosine phosphorylated after engagement of α4β1 and α5β1 integrins on human T-lymphoblastic cells. Cell. Immunol. **161**:8–13.

Ogata, T., Yamakawa, M., Imai, Y., and Takahashi, T. (1996). Follicular dendritic cells adhere to fibronectin and laminin fibers via their respective receptors. Blood **88**:2995–3003.

Oppenheimer-Marks, N., Davis, L.S., Bogue, D.T., Ramberg, J., and Lipsky, P.E. (1991). Differential utilization of ICAM-1 and VCAM-1 during the adhesion and transendothelial migration of human T lymphocytes. J. Immunol. **147**:2913–2921.

Oppenheimer-Marks, N., Davis, L.S., and Lipsky, P.E. (1990). Human T lymphocyte adhesion to endothelial cells and transendothelial migration: alteration of receptor use relates to the activation status of both the T cell and the endothelial cell. J. Immunol. **145**:140–148.

Petruzzelli, L., Takami, M., and Herrera, R. (1996). Adhesion through the interaction of lymphocyte function- associated antigen-1 with intracellular adhesion molecule- 1 induces tyrosine phosphorylation of $p130^{cas}$ and its association with c-CrkII. J. Biol. Chem. **271**:7796–7801.

Porter, J.C. and Hogg, N. (1997). Integrin cross talk: activation of lymphocyte function-associated antigen-1 on human T cells alters α4β1- and α5β1- mediated function. J. Cell Biol. **138**:1437–1447.

Porter, J.C. and Hogg, N. (1998). Integrins take partners: cross-talk between integrins and other membrane receptors. Trends Cell Biol. **8**:390–396.

Ramesh, N., Antón, I.M., Martinez-Quiles, N., and Geha, R.S. (1999). Waltzing with WASP. Trends Cell Biol. **9**:15–19.

Reif, K. and Cantrell, D.A. (1998). Networking Rho family GTPases in lymphocytes. Immunity **8**:395–401.

Roach, T.I., Slater, S.E., White, L.S., Zhang, X.L., Majerus, P.W., Brown, E.J., and Thomas, M.L. (1998). The protein tyrosine phosphatase SHP-1 regulates integrin-mediated adhesion of macrophages. Curr. Biol. **8**:1035–1038.

Romanic, A.M. and Madri, J.A. (1994). The induction of 72-kD gelatinase in T cells upon adhesion to endothelial cells is VCAM-1 dependent. J. Cell Biol. **125**:1165–1178.

Rot, A., Hub, E., Middleton, J., Pons, F., Rabeck, C., Thierer, K., Wintle, J., Wolff, B., Zsak, M., and Dukor, P. (1996). Some aspects of IL-8 pathophysiology. Chemokine interaction with endothelial cells. J. Leukocyte Biol. **59**:39–44.

Rozdzial, M.M., Malissen, B., and Finkel, T.H. (1995). Tyrosine-phosphorylated T cell receptor ζ chain associates with the actin cytoskeleton upon activation of mature T lymphocytes. Immunity 3:623–633.

Rozdzial, M.M., Pleiman, C.M., Cambier, J.C., and Finkel, T.H. (1998). $pp56^{Lck}$ mediates TCR ζ-chain binding to the microfilament cytoskeleton. J. Immunol. **161**:5491–5499.

Schimmenti, L.A., Yan, H.-C:, Madri, J.A., and Albelda, S.M. (1992). Platelet endothelial cell adhesion molecule, PECAM-1, modulates cell migration. J. Cell. Physiol. **153**:417–428.

Schlaepfer, D.D., Hauck, C.R., and Sieg, D.J. (1999). Signaling through focal adhesion kinase. Prog. Biophys. Mol. Biol. **71**:435–478.

Schwartz, M.A., Schaller, M.D., and Ginsberg, M.H. (1995). Integrins: emerging paradigms of signal transduction. Annu. Rev. Cell Biol. **11**:549–599.

Sedwick, C.E., Morgan, M.M., Jusino, L., Cannon, J.L., Miller, J., and Burkhardt, J.K. (1999). TCR, LFA-1, and CD28 play unique and complementary roles in signaling T cell cytoskeletal reorganization. J. Immunol. **162**:1367–1375.

Sells, M.A., Knaus, U.G., Bagrodia, S., Ambrose, D.M., Bokoch, G.M., and Chernoff, J. (1997). Human p21-activated kinase (Pakl) regulates actin organization in mammalian cells. Curr. Biol. 7, 202–210.

Serrador, J.M., Nieto, M., Alonso-Lebrero, J.L., Del Pozo, M.A., Calvo, J., Furthmayr, H., Schwartz-Albiez, R., Lozano, F., González-Amaro, R., Sánchez-Mateos, P., and Sánchez-Madrid, F. (1998). CD43 interacts with moesin and ezrin and regulates its redistribution to the uropods of T lymphocytes at the cell-cell contacts. Blood **91**:4632–4644.

Serrador, J.M., Nieto, M., and Sanchez-Madrid, F. (1999). Cytoskeletal rearrangement during migration and activation of T lymphocytes. Trends Cell Biol. **9**:228–232.

Shaw, A.S. and Dustin, M.L. (1997). Making the T cell go the distance: a topological view of T cell activation. Immunity **6**:361–369.

Shaw, L.M., Rabinovitz, I., Wang, H.H.F., Toker, A., and Mercurio, A.M. (1997). Activation of phosphoinositide 3-OH kinase by the α6β4 integrin promotes carcinoma invasion. Cell **91**:949–960.

Shaw, S., Luce, G.E.G., Quinones, R., Gress, R.E., Springer, T.A., and Sanders, M.E. (1986). Two antigen-independent adhesion pathways used by human cytotoxic T cell clones. Nature **323**:262–264.

Shenoi, H., Seavitt, J., Zheleznyak, A., Thomas, M.L., and Brown, E.J. (1999). Regulation of integrin-mediated T cell adhesion by the transmembrane tyrosine phosphatase CD45. J. Immunol. **162**:7120–7127.

Shimizu, Y. and Hunt, S.W., III (1996). Regulating integrin-mediated adhesion: one more function for PI 3-kinase? Immunol. Today **17**:565–573.

Shimizu, Y., Mobley, J.L., Finkelstein, L.D., and Chan, A.S.H. (1995). A role for phosphatidylinositol 3-kinase in the regulation of β1 integrin activity by the CD2 antigen. J. Cell Biol. **131**:1867–1880.

Shimizu, Y., Rose, D.M., and Ginsberg, M.H. (1999). Integrins and the immune response. Adv. Immunol. **72**:325–380.

Shimizu, Y., van Seventer, G.A., Ennis, E., Newman, W., Horgan, K.J., and Shaw, S. (1992). Crosslinking of the T cell-specific accessory molecules CD7 and CD28 modulates T cell adhesion. J. Exp. Med. **175**:577–582.

Shimizu, Y., van Seventer, G.A., Horgan, K.J., and Shaw, S. (1990a). Costimulation of proliferative responses of resting CD4+ T cells by the interaction of VLA-4 and VLA-5 with fibronectin or VLA-6 with laminin. J. Immunol. **145**:59–67.

Shimizu, Y., van Seventer, G.A., Horgan, K.J., and Shaw, S. (1990b). Regulated expression and binding of three VLA (β1) integrin receptors on T cells. Nature **345**:250–253.

Springer, T.A. (1995). Traffic signals on endothelium for lymphocyte recirculation and leukocyte emigration. Annu. Rev. Physiol. **57**:827–872.

Sriramarao, P., Von Andrian, U.H., Butcher, E.C., Bourdon, M.A., and Broide, D.H. (1994). L-selectin and very late antigen-4 integrin promote eosinophil rolling at physiological shear rates in vivo. J. Immunol. **153**:4238–4246.

Steeber, D.A., Engel, P., Miller, A.S., Sheetz, M.P., and Tedder, T.F. (1997). Ligation of L-selectin through conserved regions within the lectin domain activates signal transduction pathways and integrin function in human, mouse, and rat leukocytes. J. Immunol. **159**:952–963.

Stewart, M.P., Cabañas, C., and Hogg, N. (1996). T cell adhesion to intercellular adhesion molecule-1 (ICAM- 1) is controlled by cell spreading and the activation of integrin LFA-1. J. Immunol. **156**:1810–1817.

Stulnig, T.M., Berger, M., Sigmund, T., Raederstorff, D., Stockinger, H., and Waldhäusl, W. (1999). Polyunsaturated fatty acids inhibit T cell signal transduction by modification of detergent-insoluble membrane domains. J. Cell Biol. **143**:637–644.

Szekanecz, Z., Humphries, M.J., and Ager, A. (1992). Lymphocyte adhesion to high endothelium is mediated by two $\beta_1$ integrin receptors for fibronectin, $\alpha_4\beta_1$ and $\alpha_5\beta_1$. J. Cell Sci. **101**:885–894.

Tamura, M., Gu, J., Matusmoto, K., Aota, S., Parsons, R., and Yamada, K.M. (1998). Inhibition of cell migration, spreading, and focal adhesions by tumor suppressor PTEN. Science **280**:1614–1617.

Tanaka, Y., Adams, D.H., Hubscher, S., Hirano, H., Siebenlist, U., and Shaw, S. (1993a). T-cell adhesion induced by proteoglycan-immobilized cytokine MIP-1β. Nature **361**:79–82.

Tanaka, Y., Adams, D.H., and Shaw, S. (1993b). Proteoglycans on endothelial cells present adhesion-inducing cytokines to leukocytes. Immunol. Today **14**:111–115.

Tanaka, Y., Albelda, S.M., Horgan, K.J., van Seventer, G.A., Shimizu, Y., Newman, W., Hallam, J., Newman, P.J., Buck, C.A., and Shaw, S. (1992). CD31 expressed on distinctive T cell subsets is a preferential amplifier of β1 integrin-mediated adhesion. J. Exp. Med. **176**:245–253.

Udagawa, T., Woodside, D.G., and McIntyre, B.W. (1996). $\alpha_4\beta_1$ (CD49d/CD29) integrin costimulation of human T cells enhances transcription factor and cytokine induction in the absence of altered sensitivity to anti-CD3 stimulation. J. Immunol. **157**:1965–1972.

van Kooyk, Y., van de Wiel-van Kemenade, P., Weder, P., Kuijpers, T.W., and Figdor, C.G. (1989). Enhancement of LFA-1-mediated cell adhesion by triggering through CD2 or CD3 on T lymphocytes. Nature **342**:811–813.

van Seventer, G.A., Shimizu, Y., Horgan, K.J., and Shaw, S. (1990). The LFA-1 ligand ICAM-1 provides an important costimulatory signal for T cell receptor-mediated activation of resting T cells. J. Immunol. **144**:4579–4586.

Viola, A., Schroeder, S., Sakakibara, Y., and Lanzavecchia, A. (1999). T lymphocyte costimulation mediated by reorganization of membrane microdomains. Science **283**:680–682.

Ward, S.G., Bacon, K., and Westwick, J. (1998). Chemokines and T lymphocytes: more than an attraction. Immunity **9**:1–11.

Wardenburg, J.B., Pappu, R., Bu, J.Y., Mayer, B., Chernoff, J., Straus, D., and Chan, A.C. (1998). Regulation of PAK activation and the T cell cytoskeleton by the linker protein SLP-76. Immunity **9**:607–616.

Weerasinghe, D., McHugh, K.P., Ross, F.P., Brown, E.J., Gisler, R.H., and Imhof, B.A. (1998). A role for the αvβ3 integrin in the transmigration of monocytes. J. Cell Biol. **142**:595–607.

Willimann, K., Legler, D.F., Loetscher, M., Roos, R.S., Delgado, M.B., Clark-Lewis, I., Baggiolini, M., and Moser, B. (1998). The chemokine SLC is expressed in T cell areas of lymph nodes and mucosal lymphoid tissues and attracts activated T cells via CCR7. Eur. J. Immunol. **28**:2025–2034.

Witt, D.P. and Lander, A.D. (1994). Differential binding of chemokines to glycosaminoglycan subpopulations. Curr. Biol. **4**:394–400.

Xavier, R., Brennan, T., Li, Q.Q., McCormack, C., and Seed, B. (1998). Membrane compartmentation is required for efficient T cell activation. Immunity **8**:723–732.

Zell, T., Hunt, S.W., III, Finkelstein, L.D., and Shimizu, Y. (1996). CD28-mediated upregulation of b1 integrin-mediated adhesion involves phosphatidylinositol 3-kinase. J. Immunol. **156**:883–886.

Zell, T., Kivens, W.J., Kellermann, S.-A., and Shimizu, Y. (1998a). Regulation of integrin-mediated cell adhesion by T cell activation: points of convergence and divergence. Immunol. Res. **20**:127-145.

Zell, T., Warden, C.S., Chan, A.S.H., Cook, M.E., Dell, C.L., Hunt, S.W., III, and Shimizu, Y. (1998b). Regulation of β1-integrin-mediated adhesion by the Cbl adapter protein. Curr. Biol. **8**:814–822.

Zhang, W.G., Trible, R.P., and Samelson, L.E. (1998). LAT palmitoylation: its essential role in membrane microdomain targeting and tyrosine phosphorylation during T cell activation. Immunity **9**:239–246.

Zhang, X.A. and Hemler, M.E. (1999). Interaction of the integrin $\beta_1$ cytoplasmic domain with ICAP-1 protein. J. Biol. Chem. **274**:11–19.

